# Air Pollution of Nature Reserves near Cities in Russia

**DOI:** 10.1155/2020/9148416

**Published:** 2020-06-03

**Authors:** Aleksei Kholodov, Kirill Golokhvast

**Affiliations:** ^1^Far East Geological Institute, Far Eastern Branch of Russian Academy of Sciences, Vladivostok, Russia; ^2^Far Eastern Federal University, Vladivostok, Russia; ^3^N. I. Vavilov All-Russian Institute of Plant Genetic Resources, Saint-Petersburg, Russia; ^4^Pacific Geographical Institute, Far Eastern Branch of Russian Academy of Sciences, Vladivostok, Russia

## Abstract

Today, protected natural areas cover about 15% of the Earth's land. These areas by definition are supposed to be free of pollution; they nevertheless suffer from the effects of aerial transport of anthropogenic polluting substances. In this study, we evaluated the impact of settlements on protected natural areas to determine the optimal distance beyond which the anthropogenic influence would be minimal. For this purpose, we analyzed the particle size distribution and the content of metals in fresh snow samples collected in the Bastak Nature Reserve and the neighboring Birobidzhan city (Russian Federation). Both sites contained comparable proportions of PM_10_ and contents of heavy metals, which points to the transportation of air pollutants from the city to the reserve. The results of the analysis were summarized and compared with the available data on other nature reserves and nearby populated localities. Based on the research data, pollutant emissions should be decreased for cities that are closer than 50 km to nature reserves. Moreover, authorities should take into consideration atmospheric factors and distance to the nearest settlement when establishing new protected natural areas.

## 1. Introduction

Air pollution is a global problem as pollutants can be transported over long distances with the wind [1]. One of the more complex components of air polluting substances is particulate matter (PM), which is a mixture of multiple chemicals usually formed through chemical and physical transformations [2]. The sources of PM may be natural, such as weathering of rocks, forest fires, or the results of human activities, such as road traffic emissions, combustion of fuels, and emissions from industrial and agricultural enterprises [3]. Generally, the characteristics of airborne PM depend on their source, origin, and weather condition in a geographical area. However, particles with the aerodynamic diameter less than 10 *μ*m (PM_10_) can be transferred to distances up to tens of thousands kilometers crossing national borders [4, 5]. Fine particles (PM_2.5_) are capable of travelling larger distances and staying in the air longer [6]. There are studies dedicated to temporal and spatial distribution characteristics of atmospheric particulate matter, especially in areas suffering from significant environmental challenges [7].

As particulate matter travels large distances it also impacts protected natural areas. Protected areas are defined as designated geographical spaces, recognized, dedicated, and managed, through legal or other effective means, to achieve the long-term conservation of nature with associated ecosystem services and cultural values [8]. According to the World Database on Protected Areas (WDPA), there were 238,563 designated protected areas worldwide in 2018 [9]. Most areas are on land, collectively protecting over 20 million km^2^, equivalent to 14.9% of the Earth's land surface.

In Russian Federation, the total area of protected natural areas is steadily increasing in recent years: from 2.06 mln km^2^ in 2015 to 2.37 mln km^2^ in 2018 (13.9% of the total country's area) [10, 11]. According to another source, the total area of protected land reaches 1,641,401 km^2^ (9.73% of the country's area [12], but this discrepancy is due to the difference in methodologies and datasets used in calculation. Out of the total of 11.8 thousand protected areas in Russia, there are five categories of Federal protected areas: state nature reserves (or zapovedniks), national parks, natural parks, state nature-sanctuaries, and natural landmarks [13]. Among these, state nature reserves are the largest category in terms of area. In 2018, there were 110 state nature reserves with a total area of 345 thousand km^2^ in Russian Federation [11].

Protected natural areas by definition are established to be free of man-caused activities and, hence, of pollution. They provide ecosystem benefits for human health and climate change adaptation [14]. However, studies show that increased concentrations of air pollutants in world's natural parks raise serious concern [15–18]. In developed countries, there are national programs for monitoring atmospheric pollution in nature parks (for example, the air quality monitoring program of the US National Park Service: https://www.nps.gov/subjects/air/air-monitoring.htm).

Snow precipitation is often used to assess the environmental air pollution [19]. Snowflakes absorb more air pollutants than rain drops due to their larger surface area and slower deposition speed [20]. Also, snow sampling may be considered easier way to assess the ambient air pollution in remote areas of nature reserves compared to installing and maintaining stationary air samplers. Various techniques are applied to studying the particles in melted snow, including particle size analysis, mass spectrometry, and electron microscopy to name a few [21], with some methods capable of characterizing particles as fine as 10 nm [22].

In this study we evaluated the impact of settlements on protected natural areas to determine the optimal distance beyond which the anthropogenic influence would be minimal. For this purpose we analyzed the particulate matter in fresh snow samples collected in the Bastak Nature Reserve and the neighboring Birobidzhan city (Russian Federation) and compared the results with available data on other nature reserves and nearby populated localities.

## 2. Materials and Methods

The Bastak Nature Reserve, with the total area of approximately 92 hectares, was founded in 1997 to protect the ecosystems of the Northern Amur River region (The Jewish Autonomous Oblast, Russian Federation). Bastak mainly includes pine and linden forests with rich flora and fauna. This is the only specially protected natural area of Federal importance in the region. The reserve is located 15 km north of Birobidzhan city.

Birobidzhan is the administrative, economic, and cultural center of the Jewish Autonomous Oblast. It is a medium-size city with the total area of 150 km^2^ and a population of 73 thousand people. Birobidzhan is an industrial city with about 100 stationary air pollution sources and an intensive transport network that affects the environment [23, 24].

**Figure 1:**
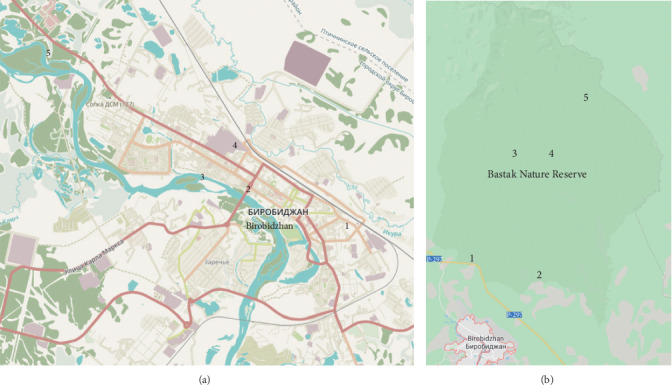
A schematic map of snow sampling points in (a) Birobidzhan city and (b) the Bastak Nature Reserve.

We collected and analyzed snow samples according to our patented method [25]. Snow samples were collected from 1 m^2^ area into 3-liter plastic containers prewashed with distilled water. Only the top layer of fresh snow was collected during or immediately after the snowfall to exclude the secondary pollution of settled snow. The samples were collected in three series at ten sampling points: five in Birobidzhan city (at November 25, 2013; February 2, 2014; and February 28, 2014) and five in the Bastak Nature Reserve (at November 20, 2013; January 29, 2014; and February 28, 2014). Snow sampling points in the city were located near major pollution sources (main roads, heat and power plant, thermoelectric plant–points G1-G4), and a reference point was located in the local forest (point G5). In the Bastak Nature Reserve, only one sample point was located near the highway (B1), while other points were located at a considerable distance from pollution sources. The location of sampling points is shown in Figure 1.

Particle size distribution of suspended particles in melted snow was measured using the Analysette 22 NanoTec plus laser particle sizer (Fritsch GmbH, Germany) with Fritch MaS software. The samples (60 ml) were diluted with 150 ml of distilled water before the analysis. The measurements were run at the settings of quartz/water at 20°C. The detection range was 0.008–2000 *μ*m.

The content of metals was measured using the Element XR high-resolution mass spectrometer (Thermo Fisher Scientific, USA). The liquid samples were stored at a temperature of 4°C and analyzed according to the method TsV 3.18.05–2005 (ZAO Centr Issledovania *i* Kontrolya vody, Saint-Petersburg, Russian Federation).

The results were compared by one-way analysis of variance using the pairwise multiple comparison procedures (the Holm-Sidak method) or Dunnett's Multiple Comparisons to a control and Student's unpaired *t*-test with Welch's correction for unequal variances. The results of metal content analysis are presented as means ± SE. *P* values of <0.05 were considered to be statistically significant.

## 3. Results

**Figure 2:**
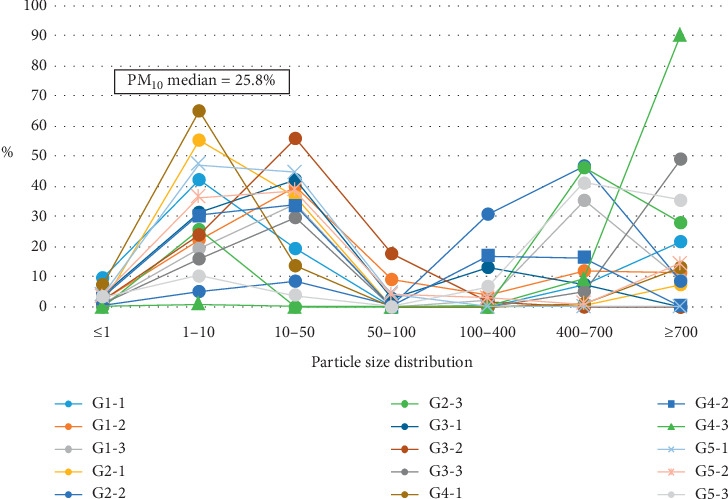
Particle size distribution graph of particulate matter in Birobidzhan city.

**Figure 3:**
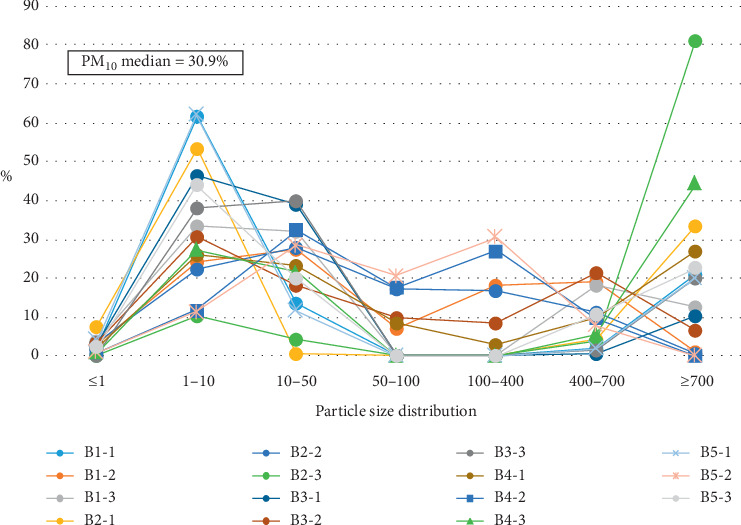
Particle size distribution graph of particulate matter in the Bastak Nature Reserve.

Similar to our previous research [26], we classified all suspended particles into seven groups: (1) 0.1–1 *μ*m (PM_1_), (2) 1–10 *μ*m (PM_10_), (3) 10–50 *μ*m, (4) 50–100 *μ*m, (5) 100–400 *μ*m, (6) 400–700 *μ*m, and (7) ≥ 700 *μ*m. Particles in melted snow samples collected during this study were divided into these groups during the particle size analysis (Tables 1 and 2, Figures 2 and 3).

According to our results (Table 1, Figure 2), ambient air in Birobidzhan city contains a significant percentage of particles with aerodynamic diameter less than 10 *μ*m, potentially hazardous to human health, mainly, the respiratory tract [27]. As previously shown, many urban air particles with aerodynamic diameter of approximately 2.5–5 *μ*m are the products of motor vehicle exhaust—soot, ashes, and metal-containing particles [28, 29]. The highest proportion of PM_10_ (64.9%) was observed in vicinity of the local thermal power station (point G4).

Unexpectedly, according to the particle size distribution data in Table 2 and Figure 3, the proportion of PM_10_ particles in the Bastak Nature Reserve is comparable and in some instances higher than that in the nearby city. One of the samples collected at a location furthest from Birobidzhan city (point B5) contained 62% of PM_10_.

According to the data of mass spectrometry analysis, heavy metals were present in samples both from Birobidzhan city and the Bastak reserve in approximately equal quantities (Table 3). An increased content of Cr was detected in the samples collected in Birobidzhan, and also, we found single cases of increased Ni and Zn content in samples from both study areas. The presence of these metals in the reserve is most likely due to their transport from the pollution source located at a 15 km distance.

## 4. Discussion

Based on the previous studies, the ambient air pollution in Birobidzhan is caused by about one hundred of stationary air pollution sources and by automobile transport [23, 24]. Industrial enterprises are mainly agglomerated in the western and north-western districts of the city, while Birobidzhan's thermal power station is in the city center. Another pollution source is the Trans-Siberian railway track running across the city.

The authors have been studying the composition and particle size of Birobidzhan's atmospheric particulate matter and its impact on the Bastak Nature Reserve for several years [30, 31]. Microparticles of both natural and anthropogenic origin were observed in the air of the nature reserve. Unfortunately, the state of the environment has not improved over several years of measurements.

In general, all Russian nature reserves employ environmental monitoring routines, which include the assessment of the quality of air, water, soil, and study of biological communities. Atmospheric pollution monitoring routines include many parameters (mostly similar to parameters controlled in major cities), but in-depth studies of particulate matter are usually not conducted. In Table 4, we summed up the basic data on the impact of settlements on the atmospheric environment of several nature reserves in Russian Federation compiled from our own research and other sources. The air pollution level was mainly assessed by studying particulate matter, its particle size distribution, chemical composition, and electron microscopy data.

As is shown in Table 4, there are at least several populated localities within a minimum distance (10–15 km) from protected natural areas. The level of impact on the environment depends on the industrial development of a city and the number of pollution sources within it. It is obvious that the distance to the reserve is not the most important factor in determining the level of pollution. However, when nature reserves are established in the immediate vicinity of a city with a minimal buffer zone, it is impossible to avoid air pollution.

There are several approaches to buffer zones surrounding protected areas. Most notably, the area-oriented approach involves zoning (mapping) of protected areas and their surroundings based on scientific data and the process-oriented approach attempts to reduce conflicts among stakeholders in protected areas through agreement and compromise [41]. Whatever the approach, many research studies and the UNESCO official position support the significance of buffer zones as the key element in the natural reserves concept [42]. However, in terms of more effective and consistent protection of nature reserves and other protected areas, the governing law should take into account the location of the newly created reserves and their distance from pollution sources. Decisions on the area of the buffer zone and position of the reserve should be based on the best available scientific evidence, and any natural reserve should have its own necessary width of buffer zone.

Despite the fact that the main sources of pollution are located outside the reserves, legislation in Russia and worldwide deals with limiting human activity inside protected areas. This contradicts the idea that a reserve is limited not only by its protected area but also by “the sphere of influence of the biosphere reserve” [43]. The buffer zone should be a zone of common interests where authorities try to keep a balance between economic necessity and a duty to protect the environment.

According to the Russian legislation the buffer zone around a nature reserve created to prevent adverse effects of anthropogenic factors should be at least 1 km. However, this width is not enough to mitigate air pollution. More strict but realistic measures should be taken in cases where the emission of air pollutants from city sources is very intensive. There are two options in the Russian environmental legislation: close down the source of pollution or impose a fine. Neither option resolves the problem and both create additional tension between the interests of the economy and the needs of environmental protection [44]. In our opinion, it is essential to develop and realize a plan for future reduction of pollutants emission. This measure would contribute to the idea of cooperation and finding a balance between anthropogenic activities and nature preservation. Accordingly, air pollution monitoring studies should continue to provide comprehensive data for future legislative decisions.

## 5. Conclusions

In this study, we evaluated the correlation between the distance of some populated localities to protected natural areas in Russian Federation in terms of air pollution with particulate matter based on our research and available data. As shown on the example of the impact of Birobidzhan city on the Bastak Nature Reserve, protected natural areas suffer from anthropogenic influence. It is necessary to improve the air monitoring system in Russian nature reserves and correlate it more closely with the data on atmospheric pollution in urban areas.

The short distance between cities and nature reserves is beneficial for the cities but can be detrimental for ecosystems in protected natural areas. There are two possible legal actions that could deal with the issue: on the one hand, reserves could be moved further away from the cities, and on the other hand, cities could be obliged to decrease air pollutants emissions. In our opinion, based on the research data, emissions would need to be decreased for cities that are closer than 50 km to nature reserves. Moreover, authorities should take into consideration atmospheric factors and distance to the nearest settlement when establishing new protected natural areas.

## Figures and Tables

**Table 1:** Particle size distribution in snow samples collected in Birobidzhan city.

PM fraction (*μ*m)	Distribution of PM in snow samples (%)														
	G1			G2			G3			G4			G5		
	1	2	3	1	2	3	1	2	3	1	2	3	1	2	3
≤1	9.7	2.3	1	0.8	0.6	0	4.1	2.1	0.9	7.5	2.9	0	3.8	3.3	3.1
1–10	41.9	22.1	19	55.1	4.7	25.8	31	23.6	15.7	64.9	30.2	0.9	47.2	36.3	10.2
10–50	19.5	39.6	33.9	36.7	8.2	0	42.1	55.7	29.5	13.5	33.6	0	44.6	38.7	3.6
50–100	0	9.2	0	0	0.5	0	2.7	17.3	0	0	0	0	4.4	3.9	0
100–400	0	3.7	1.9	0	30.8	0.3	12.7	1.3	0	0	16.8	0	0	2.7	6.5
400–700	7.5	11.8	35.5	0.3	46.7	45.9	7.4	0	4.9	0.9	16.2	9	0	0.8	41.1
≥700	21.4	11.3	8.7	7.1	8.5	28	0	0	49	13.2	0.3	90.1	0	14.3	35.5
Mean diameter	234.0	183.3	277.6	74.4	410.2	492.8	79.6	29.0	485.2	130.9	138.1	892.8	16.4	152.6	553.6

**Table 2:** Particle size distribution in snow samples collected in the Bastak Nature Reserve.

PM fraction (*μ*m)	Distribution of PM in snow samples (%)														
	B1			B2			B3			B4			B5		
	1	2	3	1	2	3	1	2	3	1	2	3	1	2	3
≤1	2.2	3	3.9	7.6	3.4	0.1	3.1	3.6	0.2	2	1.1	1	4.3	1.1	2.5
1–10	61.6	24.3	33.4	53.6	22.2	10.3	46.6	30.9	37.9	25.9	11.9	27.3	62	11.4	44
10–50	13.4	27.4	31.9	0.9	28.1	4.5	39.2	18.3	40	23.5	32.4	21.5	11.7	28.5	20
50–100	0	7	0	0	17.4	0	0	10	0	8.6	17.6	0	0	20.6	0
100–400	0	18.2	0	0	17	0	0	8.7	0	3.1	27.1	0	0	30.5	0
400–700	1.9	19	18.2	4.2	11.3	4.1	0.5	21.6	1.8	9.9	9.9	5.6	1.9	7.9	10.7
≥700	20.9	1	12.6	33.7	0.6	81	10.6	6.9	20.1	27	0	44.6	20.1	0	22.8
Mean diameter (*μ*m)	207.2	154.5	215.3	332.2	117.5	800.8	110.2	257.7	201.9	311.0	137.9	445.5	199.6	128.7	262.6

**Table 3:** Results of mass spectrometry of the snow samples collected in Birobidzhan city and the Bastak Nature Reserve, *μ*g/L.

	Al	Cr	Fe	Ni	Cu	Zn	Cd	Ba	Pb	As

*Birobidzhan city*										
G1	4.15 ± 1.24	1.49 ± 0.45	30.53 ± 9.16	0.37 ± 0.11	1.1 ± 0.33	23.93 ± 7.18	0.011 ± 0.003	3.38 ± 1.0169	0.51 ± 0.15	0.69 ± 0.21
G2	1.5 ± 0.45	1.78 ± 0.53	7.3 ± 2.2	0.38 ± 0.11	1.10 ± 0.33	14.82 ± 4.45	0.008 ± 0.002	2.02 ± 0.60	0.58 ± 0.17	0.60 ± 0.18
G3	1.36 ± 0.41	2.23 ± 0.67	3.13 ± 0.94	0.35 ± 0.11	0.71 ± 0.21	27.14 ± 8.14	0.012 ± 0.003	4.27 ± 1.28	1.57 ± 0.47	0.56 ± 0.17
G4	2.18 ± 0.65	3.22 ± 0.96	3.92 ± 1.18	0.33 ± 0.09	0.73 ± 0.22	11.36 ± 3.41	0.005 ± 0.002	1.66 ± 0.5	0.34 ± 0.103	0.39 ± 0.12
G5	4.58 ± 1.37	2.80 ± 0.84	0.85 ± 0.25	0.92 ± 0.27	0.42 ± 0.12	19.52 ± 5.86	0.004 ± 0.001	1.81 ± 0.54	0.17 ± 0.05	0.47 ± 0.14

*The Bastak Nature Reserve*										
B1	1.32 ± 0.39	0.04 ± 0.01	1.86 ± 0.56	0.26 ± 0.08	0.57 ± 0.17	7.30 ± 2.19	0.003 ± 0.001	0.58 ± 0.17	0.15 ± 0.04	0.13 ± 0.04
B2	2.50 ± 0.75	0.04 ± 0.01	2.71 ± 0.81	0.21 ± 0.06	0.54 ± 0.16	8.21 ± 2.46	0.003 ± 0.001	0.68 ± 0.21	0.13 ± 0.04	0.18 ± 0.05
B3	0.75 ± 0.22	0.14 ± 0.04	2.03 ± 0.61	11.97 ± 3.59	2.65 ± 0.79	20.56 ± 6.17	0.0013 ± 0.0004	0.71 ± 0.21	0.02 ± 0.01	0.09 ± 0.02
B4	2.24 ± 0.67	0.27 ± 0.08	1.05 ± 0.31	0.56 ± 0.17	0.33 ± 0.1	19.48 ± 5.84	0.004 ± 0.0013	2.49 ± 0.75	0.14 ± 0.04	0.23 ± 0.07
B5	0.70 ± 0.21	0.03 ± 0.01	0.72 ± 0.22	1.14 ± 0.34	0.58 ± 0.17	8.66 ± 2.59	0.0013 ± 0.0004	0.25 ± 0.075	0.13 ± 0.04	0.12 ± 0.04

**Table 4:** Impact of the nearest settlements on the air of some nature reserves in Russian Federation.

Nature reserve	Nearest populated locality	Distance from the reserve to the locality (km)	Population of the locality (2018)	Air pollution level in the reserve
Norsky	Fevralsk	10	4731	Low [32]
Zeysky	Zeya	10	23 270	Low [33]
Sikhote-Alin	Terney	5	3311	Low [34, 35]
Botchinsky	Sovetskaya Gavan	120	23 816	Average [36]
Bastak	Birobidzhan	15	73 623	High ([30, 31], this study)
Lipoviy Ostrov (natural monument)	Novokuznetsk	50	553 638	Low [37]
Baikal	Baikalsk	78	12 629	Average [38]
	Irkutsk	220	623 869	
Stolby	Krasnoyarsk	15	1 090 811	Average [39]
Voronezhsky	Voronezh	47	1 047 549	Low [40]

## Data Availability

No data were used to support this study.
